# COMBATING STIGMA: AN EDUCATIONAL RESOURCE ON SUBSTANCE USE DISORDER IN OLDER ADULTS

**DOI:** 10.1093/geroni/igac059.1721

**Published:** 2022-12-20

**Authors:** Marissa Mackiewicz, Patricia Slattum, Leland Waters

**Affiliations:** Virginia Commonwealth University, Richmond, Virginia, United States; Virginia Commonwealth University, Richmond, Virginia, United States; Virginia Commonwealth University, Richmond, Virginia, United States

## Abstract

Substance Use Disorder (SUD) affects people from all walks of life and age groups. Special considerations for older adults with SUD should be made secondary to the unique pathophysiological and socio-economic complexities of aging populations. Successful SUD treatment and recovery depends on access to essential support systems comprised of interprofessional teams as well as family, friends, and caregivers. Education on SUD for healthcare providers, caregivers and the community is a key component for combating the national opioid epidemic and the stigma that can be a barrier to recovery. A ten-minute video and an accompanying discussion guide were developed to present to clinical faculty and students participating in an interprofessional care coordination wellness clinic servicing low-income older adults living independently in the community setting. The video was designed to highlight stigma and ageism facing older adults with SUD and serve as the basis for discussion among learners. The process for creating and evaluating the video and discussion guide will be shared including the role of older adults with lived experience of SUD in the process. Information will be provided on SUD as a chronic disease and the importance of interprofessional and individualized patient care in recovery. Participants will be able to identify the significance of SUD related stigma in providing and seeking care, asses the role of mental/behavioral health in SUD, and learn about treatment options for SUD. This will allow for better identification of characteristics of older adult populations that place them at greater harm from the consequences SUD.

